# Successful Treatment of Methampyrone-Induced Toxic Epidermal Necrolysis with Therapeutic Plasma Exchange

**DOI:** 10.1155/2018/2182604

**Published:** 2018-07-18

**Authors:** Santosa, A. Rumbiana, Z. Wahab, S. P. Kurniawan, R. M. Naibaho, P. Yogyartono

**Affiliations:** ^1^Division of Hematology and Medical Oncology, Department of Internal Medicine, Medical Faculty of Diponegoro University and Dr. Kariadi Hospital, Semarang, Indonesia; ^2^Department of Internal Medicine, Medical Faculty of Mulawarman University, A. M. Parikesit Hospital and A. W. Sjahranie Hospital, Tenggarong-Samarinda, Indonesia; ^3^Department of Dermatology and Venereology, Telogorejo Hospital, Semarang, Indonesia

## Abstract

The toxic epidermal necrolysis (TEN) is a severe cutaneous adverse reaction frequently caused by drug exposure. A 58-year-old male was admitted to the hospital after administration of methampyrone. He developed sloughing of the total epidermis which rapidly extended over the trunk and limbs. The presumptive diagnosis was drug-induced TEN. Despite the treatment with pulsed glucocorticoid and cyclosporine, the skin lesions extended over the entire body. Strikingly, the progression of blistering was stopped by therapeutic plasma exchange (TPE). TPE was discontinued after the signs of skin inflammation had been overcome. He recovered in 8 days of hospitalization. We present here a case of a methampyrone-induced TEN which was successfully treated with TPE.

## 1. Introduction

Toxic epidermal necrolysis (TEN) is a severe cutaneous reaction characterized by extensive epidermal necrolysis (more than 30% of body surface), fever, and mucosal tissue involvement with conjunctivitis [1]. The pathogenesis of TEN remains unknown but represents an idiosyncratic drug reaction. Most commonly implicated drugs are nonsteroidal anti-inflammatory drugs [2] with the incidence in approximately 0.9–1.4 cases/million inhabitants/year [3]. TEN has considerable morbidity and may be life-threatening in substantial cases (up to 50% mortality rate) thus should be treated in intensive care or burn unit [4].

The treatment of TEN is mostly based on suspected drug withdrawal; acute management is mainly supportive [1, 4, 5]. Due to both complex pathophysiology and lack of randomized clinical trials (RCTs), the specific treatment for TEN is not established as well as the treatment with plasma exchange—it was described only in clinical cases. In the following, we describe a case where severe TEN caused by methampyrone ingestion has been treated successfully using therapeutic plasma exchange (TPE).

## 2. Case Report

In February 2018, we treated the patient, a 58-year-old male who developed erythematous skin with severe itching and flaking presented on the entire body surface. Detailed history suggested that the patient consulted a private physician for a toothache for which he was prescribed with methampyrone 500 mg orally. After taking a single dose of the drug, he developed maculopapular and erythematous rash with itching that followed by bullous exfoliation of the skin. Past medical history included hypertension and postprimary coronary intervention in 2011. The patient has been taking aspirin 80 mg QD, amlodipine 10 mg QD, and atorvastatin 20 mg QD.

**Figure 1:**
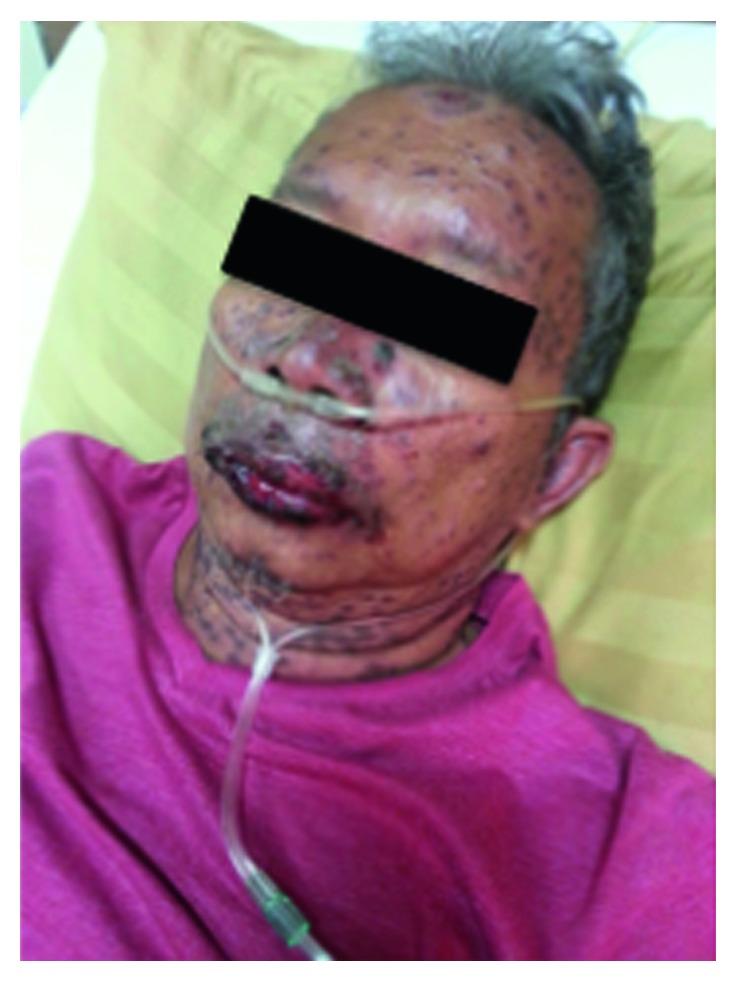
Facial and lip swelling along with conjunctivitis of the eyes in toxic epidermal necrolysis (initial presentation).

On examination, the patient was conscious and alert, but he looked weak. Hemodynamics was stable, with the respiration rate of 24x/minutes, body temperature of 37.8°C, and SpO_2_ of 97–99% while breathing suplementary oxygen with nasal cannula. There were conjunctivitis and turbid corneal in the bilateral eyes (not shown), ulceration of the mouth, and swollen lips (Figure 1). He had generalized skin erythema and irregularly shaped itchy purpuric macules. Nikolsky's sign was clearly elicited with a detachment of the epidermis from lower layers when slightly rubbed, and extension of existing bullae to the clear skin indicated an active TEN. The epidermal detachment was observed over 30% of the body surface area (BSA).

**Figure 2:**
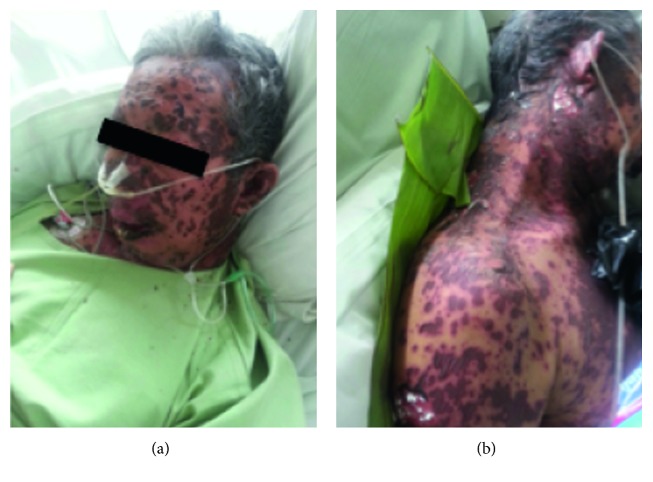
The patient's skin changes of the trunk and extremities with sloughing of the epidermis (during hospitalization). Hemorrhagic blisters evolved within days of usual treatment.

Treatment for the patient was involving replacement of fluid loss and also maintaining electrolyte imbalance and antibiotic therapy. He started methylprednisolone 125 mg TID along with cyclosporine 50 mg BID. After 2 days of hospitalization, his skin lesions did not show improvement.In turn, skin change progressions rapidly extended from 32% at hospital admission to 62% of BSA involved with 16% in grade I and 46% in grade II hemorrhagic blisters (Figure 2). The SCORTEN score [6] used to prognosticate risk for death from TEN was three in this patient with the corresponding predictive mortality rate of 35.3%.

By clinical judgement of the lack of patient's response to initial therapy, we decided to treat him with TPE. The procedure was performed using the COBE Spectra Apheresis System (Terumo BCT, Inc., Lakewood, CO) and a double membrane filtration device via central vascular access. The exact filtered plasma volume was calculated with regard to patient's weight and hematocrit level. On each exchange, about 2 L of plasma was removed at blood flow of 50 mL/minute; the replacement of fluid consisted of about 1 L of 5% albumin and 1 L of normal saline. Anticoagulant citrate dextrose solution-A (ACD-A) was used as an anticoagulant during the procedure at a ratio of 1 : 12. In addition, two grams of IV calcium gluconate was administered as prophylaxis against citrate toxicity. TPE was started on day 3 and provided every 2 days for a total of three procedures.

**Figure 3:**
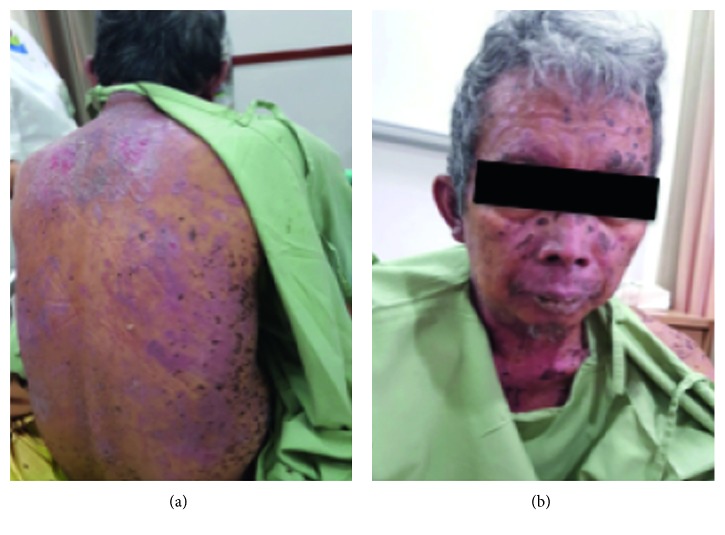
The photograph of skin lesions after 3 treatment sessions of TPE; it was taken 1 day before hospital discharge.

The patient's condition rapidly improved after the completion of the first TPE session. Blistering with extensive epidermal necrosis halted after the second and third session of TPE, and then the epidermal sheet began to dry up and the skin erosions started to heal. Rapid reepithelization occur by 1 week of the introduction of TPE (Figure 3).The patient made an uneventful recovery, and he was discharged home in 8 days of hospitalization with good condition. Proper instructions were given regarding a possible relapse and methampyrone avoidance. At the checkup, the lesions had completely disappeared.

## 3. Discussion

TEN is a rare and potentially fatal cutaneous reaction to medicaments [1, 2]. In the present case, the diagnosis of TEN is performed by characterization of lesions and distinctive clinical features as shown in figures. The causality is “probable” to the drug methampyrone with a score of seven on Naranjo causality assessment scale [7] and “probable or likely” based on the World Health Organization (WHO) scale [8].

In Indonesia, few studies have been conducted to identify the prevalence and the causative drugs of TEN [9, 10]. Two retrospective studies by Suwarsa et al. [9] and Abdullah et al. [10] reported that the drugs most frequently implicated in case of TEN were analgesic-antipyretic, although methampyrone was not included in the list of drugs associated with the incident. A large population-based case-control study covering about persons in several European countries was conducted between the years 1989 and 1992 reporting 3% versus 1% of the controls using methampyrone [11]. The potential risk of methampyrone to elicit severe skin drug eruption is considered low or uncertain [2, 11].

Methampyrone has more than 20 known metabolites [12]. In some susceptible individuals, metabolites may not be perfectly detoxified and instead act as a hapten and initiate an immunologic response that results in cellular necrosis [1, 5]. Our patient had severe TEN with 62% of BSA affected who did not respond to standard treatment. His clinical status was deteriorated. As there are no guidelines on how to treat TEN patients refractory to glucocorticoid which usually have the highest mortality, we decided to start TPE immediately.

The decision to opt TPE was encouraged by the positive effect of TEN patients (*n* = 47) reported by Yamada et al. Most of the patients (76%) in their publication were unresponsive to standard treatment before the commencement of TPE [13]. Studies from Poland [14] and the Czech Republic [15] also emphasized that the treatment of TEN with TPE could be considered in patients who were refractory to corticosteroid and intravenous immunoglobulins (IVIG). Narita et al. [16] provided evidence to support a proposed mechanism of action of TPE. Their report underlined that TPE removes pathogenic factors such as proinflammatory cytokines or unknown toxic substances from the plasma of TEN patients.

To the best of our knowledge, no published case report of TEN successfully treated with TPE has been reported in Indonesia. The American Society of Apheresis currently classifies TPE as third-line treatment for refractory TEN with only weak recommendation (grade IIB) [17]. The unavailability of plasmapheresis unit for the procedure, need for central venous access, cost-effectiveness, and proper setup are the main hurdles in opting this method of treatment.

## 4. Conclusion

Although TPE is expensive and requires venous access to be performed, this case report demonstrated rapid improvement of TEN in the older patient. TPE could be considered as an alternative therapeutic approach for severe forms of TEN in particular if initial treatments fail. TPE appears safe; however, the further prospective study would be appropriate to fully define its place in the treatment of TEN.
